# Metastatic disease after removal of a renal cell carcinoma smaller than 3 cm in a patient with Birt-Hogg-Dubé syndrome, a case report

**DOI:** 10.1007/s10689-024-00408-w

**Published:** 2024-06-20

**Authors:** L. van Riel, C. M. Kets, L. P. van Hest, F. H. Menko, B. G. Boerrigter, S. M. Franken, R. M.F. Wolthuis, H. J. Dubbink, P. J. Zondervan, R. J.A. van Moorselaar, A. C. Houweling, I. van de Beek

**Affiliations:** 1 Department of Human Genetics, Amsterdam UMC, University of Amsterdam, Amsterdam, the Netherlands; 2 Department of Human Genetics, Cancer Center Amsterdam, Amsterdam UMC, Vrije Universiteit Amsterdam, Amsterdam, the Netherlands; 3 Department of Human Genetics, Amsterdam UMC, Vrije Universiteit Amsterdam, Amsterdam, the Netherlands; 4 Department of Human Genetics, Radboud University Medical Center, Nijmegen, the Netherlands; 5 Department of Clinical Genetics, Netherlands Cancer Institute, Amsterdam, the Netherlands; 6 Department of Pulmonary Medicine, Amsterdam UMC, Vrije Universiteit Amsterdam, Amsterdam, the Netherlands; 7 Department of Dermatology, Amsterdam UMC, University of Amsterdam, Amsterdam, the Netherlands; 8 Department of Pathology, Erasmus MC, University Medical Center Rotterdam, Rotterdam, the Netherlands; 9 Department of Urology, Amsterdam UMC, University of Amsterdam, Amsterdam, the Netherlands; 10 Department of Urology, Amsterdam UMC, Vrije Universiteit Amsterdam, Amsterdam, the Netherlands

**Keywords:** Birt-Hogg-Dubé syndrome, BHD, Renal cell carcinoma, Metastasis, FLCN, Genetics

## Introduction

Birt-Hogg-Dubé syndrome (BHD, MIM #135150) is an autosomal dominant hereditary predisposition for fibrofolliculomas, pulmonary cysts, pneumothorax and renal cell carcinoma (RCC). The estimated RCC risk in BHD is 16–21% until age 70 [1, 2] and patients with BHD are advised to undergo regular renal surveillance [3]. BHD-associated RCC may be bilateral and/or multifocal and often has a chromophobe component [4, 5]. In general, BHD-associated renal tumors are considered to be of indolent behavior and therefore, treatment of renal tumors can be postponed until the tumor reaches a diameter of 3 cm (3 cm rule) [6, 7].

## Case description

**Fig. 1:**
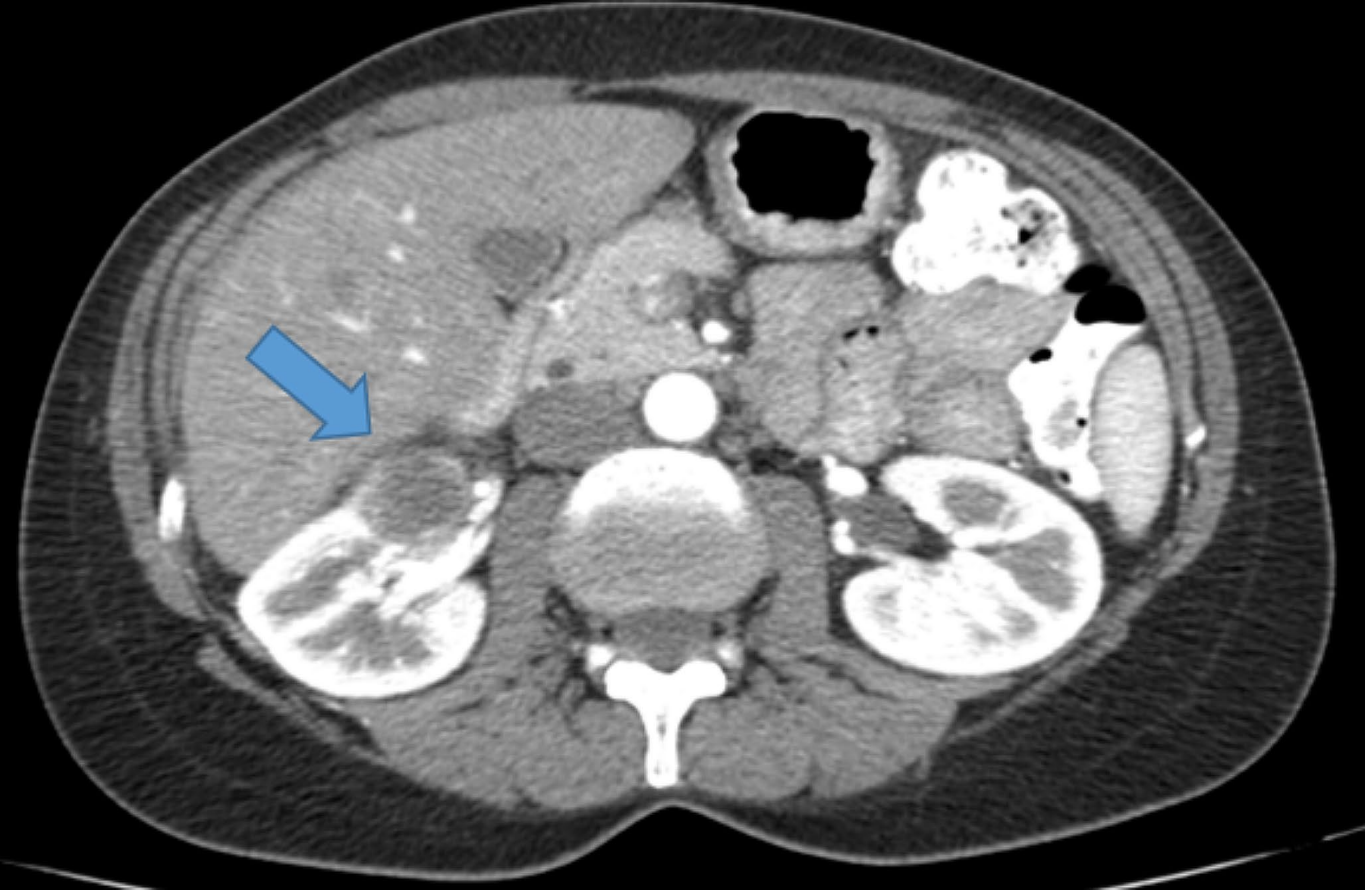
Renal tumor

A 53 year old women presented with acute back pain. Ultrasound examination showed a right-sided renal mass measuring 2.5 × 1.5 × 2 cm. On CT, the tumor had a maximal diameter of 2.8 cm (indicated by the arrow in Fig. 1).

She was initially treated by partial nephrectomy. Histological evaluation showed a 2.8 cm eosinophilic chromophobe RCC, Fuhrmann grade 2, positive for cytokeratin 7, 8 and 18, EMA, CD10, and colloidal iron and negative for vimentin. As not all resection margins were free of tumor, a radical nephrectomy was performed subsequently. No residual tumor was detected in the remaining kidney tissue. Follow-up took place according to appropriate guidelines for RCC. At age 59, six years after removal of the primary tumor, six omental metastases were identified on abdominal CT. A biopsy showed a malignancy, histologically comparable to the previously removed primary tumor (PAX8, CK7, CD117 and CD10 positive). Since the patient had no lesions in her remaining kidney, the omental lesions were considered metastases from the primary tumor diagnosed 6 years previously. Molecular testing to get extra evidence for clonality between the primary tumor and the metastases was not possible, as no suitable material of the primary tumor was available. After diagnosis of the omental metastases, the patient was treated with immunotherapy in a phase II study (4 cycles nivolumab and iplimumab followed by 14 cycles nivolumab). The metastases show minimal progression despite immunotherapy and currently she is in a wait-and-see policy.

**Fig. 2:**
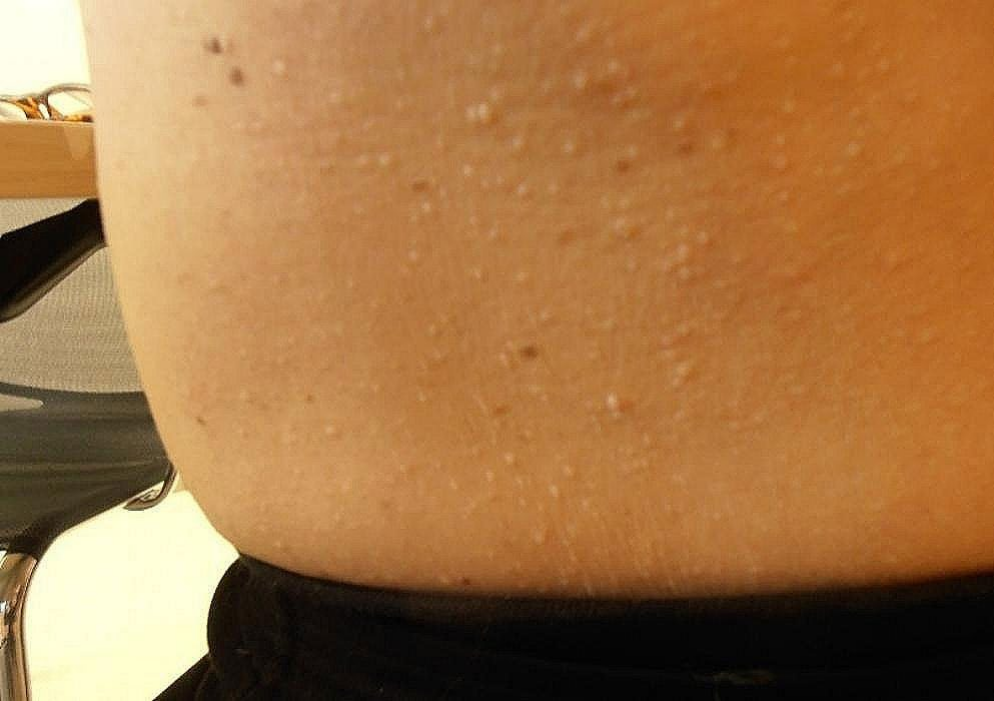
Skin lesions on abdomen

The family history was negative for RCC and pneumothorax. On physical examination, the patient had multiple small papules compatible with fibrofolliculomas (Fig. 2). They were subtle on the face, more prominent in the neck and innumerable on chest and abdomen. Genetic testing at age 59 confirmed the clinical diagnosis of BHD: A previously reported pathogenic germline variant was identified in *FLCN* (c.619-1G > A) [1]. Family members were offered genetic testing and upon detection of the *FLCN* variant, renal surveillance.

**Fig. 3:**
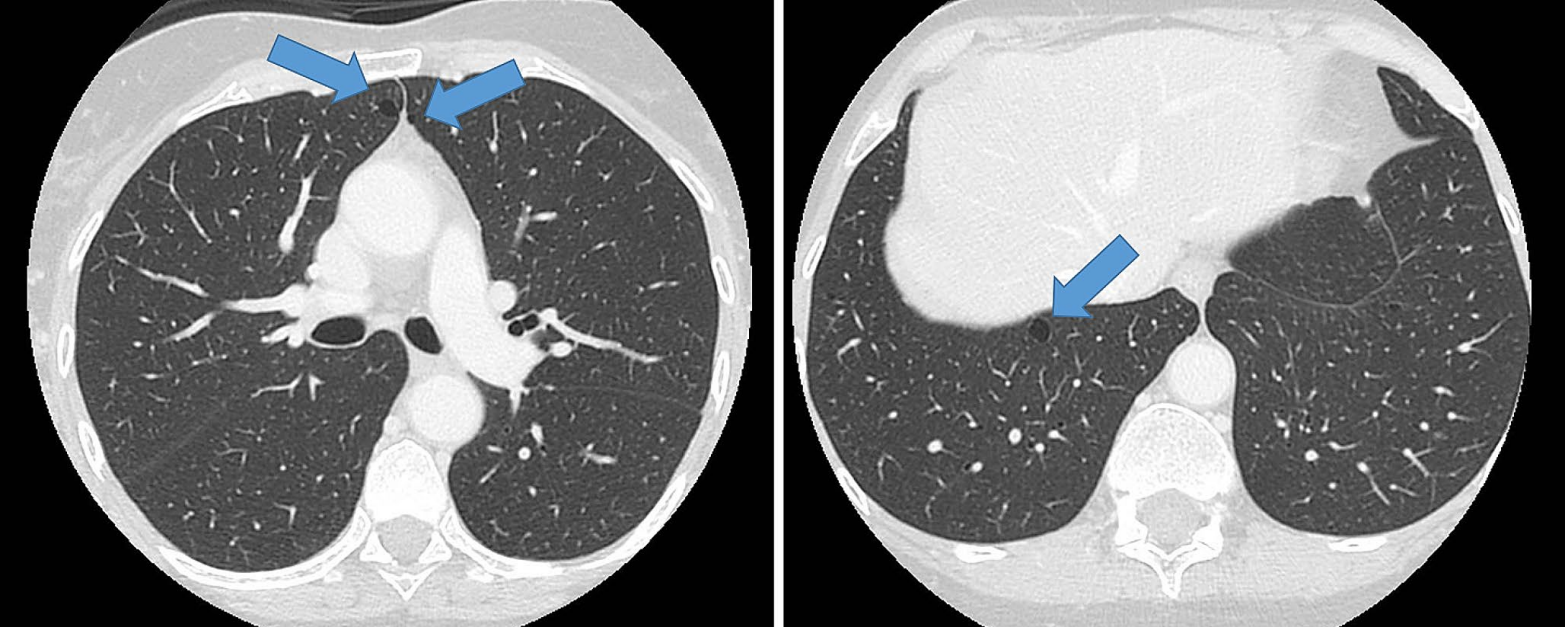
Lung cysts

Chest imaging was performed as part of staging and follow-up from age 53 onwards. Chest CT’s retrospectively showed around 10–20 bilateral thin-walled cysts, most prominent in the basal parts of the lungs, consistent with BHD (indicated by the arrows in Fig. 3, chest CT at age 60). Since these cysts were quite subtle, they had not been recognized as possibly related to BHD initially [8].

## Discussion

To the best of our knowledge, this is the first report of metastasized chromophobe RCC, after complete resection of a primary tumor smaller than 3 cm, in a patient with BHD.

In patients with BHD, the 3 cm rule is commonly applied in the treatment of renal tumors. The 3 cm rule was initially established for patients with von Hippel-Lindau disease (VHL). Two prospective studies showed that the 3 cm rule was effective in minimizing the risk of metastases while maximizing renal function in patients at risk for multifocal and bilateral RCC [9, 10]. These studies were mainly performed in patients with VHL and hereditary papillary RCC; only one patient with a chromophobe RCC and BHD was included. Nonetheless, the 3 cm rule was also implemented for BHD patients [6]. Indirect evidence that the 3 cm rule is safe in BHD is that (1) until now, to our knowledge, no reports of metastases associated with renal tumors smaller than 3 cm were described in BHD patients, and (2) the growth rate of BHD-associated renal tumors is slower than VHL-associated RCC [11]. This is in contrast to Hereditary Leiomyomatosis and Renal Cell Carcinoma (HLRCC), in which even small tumors have a high potential to metastasize [12].

Chromophobe RCC, as in our patient, is common in patients with BHD, although other histological subtypes occur as well [3]. Chromophobe RCC is less aggressive as compared to other RCC subtypes and in general rarely metastasizes [13, 14]. The omentum is an uncommon location for metastases, with the most common locations being lung, bone, liver and lymph nodes [15]. Few cases have been reported in literature of omental metastases of a primary RCC [16–19]. However, these primary tumors were not of chromophobe origin. The mechanism for peritoneal involvement in RCC is not fully understood, but it might be caused by hematogenous spread and/or direct spreading. Although probably rare, tumor seeding due to surgery also has to be taken into consideration [20–22]. However, no abnormalities were documented during the surgical procedure of the primary tumor in our patient.

Since our patient presented with metastatic chromophobe RCC, even though the primary tumor of many years ago was smaller than 3 cm on imaging and upon histological evaluation, the question arises whether applying the 3 cm rule in BHD patients is the right strategy. On one hand, it may not be justified to adjust existing screening recommendations based on exceptional observations in single cases. Most likely, the patient presented here might be a single outlier, as these are expected in screening for (inherited) diseases. For example, renal surveillance in BHD often starts at age 20 [3], but a patient with BHD and RCC at age 14 has been reported [23]. Screening guidelines aim to strike a balance between the benefits of screening and the burdens on the patient, taking into account cost-effectiveness. Thus, surveillance recommendations often do not provide complete certainty. On the other hand, metastases in smaller BHD-associated renal tumors may be more common than is currently known. For example because cases may not have been published in the literature, or these patients might not have been recognized as having BHD. Another potential explanation for the lack of data on tumors smaller than 3 cm that metastasized could be that part of the BHD-associated tumors are treated upon detection, even when smaller than 3 cm. However, it is reassuring that metastases in patients with a primary tumor smaller than 3 cm have not been reported. In two BHD cohorts combined, over 70 BHD-associated RCC were reported and the small number of tumors that metastasized were all larger than 6 cm at diagnosis [4, 5].

In conclusion, it is necessary to report experiences with screening, surveillance and treatment in this patient group. Further research in large BHD case series is required to establish whether screening recommendations should be reconsidered.

## Data Availability

No datasets were generated or analysed during the current study.

## References

[1] Houweling AC, Gijezen LM, Jonker MA, van Doorn MB, Oldenburg RA, van Spaendonck-Zwarts KY et al (2011) Renal cancer and pneumothorax risk in Birt-Hogg-Dube syndrome; an analysis of 115 FLCN mutation carriers from 35 BHD families. Br J Cancer 105(12):1912–1919 22146830 10.1038/bjc.2011.463 PMC3251884

[2] Bruinsma FJ, Dowty JG, Win AK, Goddard LC, Agrawal P, Attina D et al (2023) Update of penetrance estimates in Birt-Hogg-Dube syndrome. J Med Genet 60(4):317–326 36849229 10.1136/jmg-2022-109104

[3] Menko FH, van Steensel MA, Giraud S, Friis-Hansen L, Richard S, Ungari S et al (2009) Birt-Hogg-Dube syndrome: diagnosis and management. Lancet Oncol 10(12):1199–1206 19959076 10.1016/S1470-2045(09)70188-3

[4] Pavlovich CP, Grubb RL 3rd, Hurley K, Glenn GM, Toro J, Schmidt LS et al (2005) Evaluation and management of renal tumors in the Birt-Hogg-Dube syndrome. J Urol 173(5):1482–1486 10.1097/01.ju.0000154629.45832.30 15821464

[5] Johannesma PC, van de Beek I, van der Wel T, Reinhard R, Rozendaal L, Starink TM et al (2019) Renal imaging in 199 Dutch patients with Birt-Hogg-Dube syndrome: screening compliance and outcome. PLoS ONE 14(3):e0212952 30845233 10.1371/journal.pone.0212952 PMC6405080

[6] Barrisford GW, Singer EA, Rosner IL, Linehan WM, Bratslavsky G (2011) Familial renal cancer: molecular genetics and surgical management. Int J Surg Oncol 2011:658767 22312516 10.1155/2011/658767 PMC3263689

[7] Stamatakis L, Metwalli AR, Middelton LA, Marston Linehan W (2013) Diagnosis and management of BHD-associated kidney cancer. Fam Cancer 12(3):397–402 23703644 10.1007/s10689-013-9657-4 PMC4175415

[8] Johannesma PC, Houweling AC, Menko FH, van de Beek I, Reinhard R, Gille JJ et al (2016) Are lung cysts in renal cell cancer (RCC) patients an indication for FLCN mutation analysis? Fam Cancer 15(2):297–300 26603437 10.1007/s10689-015-9853-5 PMC4803815

[9] Herring JC, Enquist EG, Chernoff A, Linehan WM, Choyke PL, Walther MM (2001) Parenchymal sparing surgery in patients with hereditary renal cell carcinoma: 10-year experience. J Urol 165(3):777–781 11176466

[10] Walther MM, Choyke PL, Glenn G, Lyne JC, Rayford W, Venzon D, Linehan WM (1999) Renal cancer in families with hereditary renal cancer: prospective analysis of a tumor size threshold for renal parenchymal sparing surgery. J Urol 161(5):1475–1479 10210376 10.1016/s0022-5347(05)68930-6

[11] Ball MW, An JY, Gomella PT, Gautam R, Ricketts CJ, Vocke CD et al (2020) Growth rates of genetically defined renal tumors: implications for active surveillance and intervention. J Clin Oncol 38(11):1146–1153 32083993 10.1200/JCO.19.02263 PMC7145590

[12] Grubb RL 3rd, Franks ME, Toro J, Middelton L, Choyke L, Fowler S et al (2007) Hereditary leiomyomatosis and renal cell cancer: a syndrome associated with an aggressive form of inherited renal cancer. J Urol. ;177(6):2074-9; discussion 9–80 10.1016/j.juro.2007.01.155 17509289

[13] Yip SM, Ruiz Morales JM, Donskov F, Fraccon A, Basso U, Rini BI et al (2017) Outcomes of metastatic chromophobe renal cell carcinoma (chrRCC) in the targeted therapy era: results from the International Metastatic Renal Cell Cancer Database Consortium (IMDC). Kidney Cancer 1(1):41–47 30334003 10.3233/KCA-160002 PMC6179119

[14] Garje R, Elhag D, Yasin HA, Acharya L, Vaena D, Dahmoush L (2021) Comprehensive review of chromophobe renal cell carcinoma. Crit Rev Oncol Hematol 160:103287 33753250 10.1016/j.critrevonc.2021.103287

[15] Dudani S, de Velasco G, Wells JC, Gan CL, Donskov F, Porta C et al (2021) Evaluation of Clear Cell, Papillary, and Chromophobe Renal Cell Carcinoma Metastasis Sites and Association with Survival. JAMA Netw Open 4(1):e2021869 33475752 10.1001/jamanetworkopen.2020.21869 PMC7821027

[16] Acar O, Mut T, Saglican Y, Sag AA, Falay O, Selcukbiricik F et al (2016) Isolated omental metastasis of renal cell carcinoma after extraperitoneal open partial nephrectomy: a case report. Int J Surg Case Rep 21:6–11 26874583 10.1016/j.ijscr.2016.02.008 PMC4802132

[17] Staderini F, Cianchi F, Badii B, Skalamera I, Fiorenza G, Foppa C et al (2015) A unique presentation of a renal clear cell carcinoma with atypical metastases. Int J Surg Case Rep 11:29–32 25911241 10.1016/j.ijscr.2015.03.009 PMC4446666

[18] Jennison E, Wathuge GW, Gorard DA (2015) Renal cell carcinoma presenting with malignant ascites. JRSM Open 6(4):2054270415585087 25973217 10.1177/2054270415585087 PMC4429040

[19] Tartar VM, Heiken JP, McClennan BL (1991) Renal cell carcinoma presenting with diffuse peritoneal metastases: CT findings. J Comput Assist Tomogr 15(3):450–453 2026808 10.1097/00004728-199105000-00019

[20] Castillo OA, Vitagliano G (2008) Port site metastasis and tumor seeding in oncologic laparoscopic urology. Urology 71(3):372–378 18342166 10.1016/j.urology.2007.10.064

[21] Ploumidis A, Panoskaltsis T, Gavresea T, Yiannou P, Yiannakou N, Pavlakis K (2013) Tumor seeding incidentally found two years after robotic-assisted radical nephrectomy for papillary renal cell carcinoma. A case report and review of the literature. Int J Surg Case Rep 4(6):561–564 23632074 10.1016/j.ijscr.2013.03.031 PMC3650258

[22] Rodriguez Fernandez E, Cardo AL, Subira Rios D, Cancho Gil MJ, Gonzalez Garcia FJ, Herranz Amo F, Hernandez Fernandez C (2022) Peritoneal carcinomatosis after partial nephrectomy for renal cell carcinoma: our experience and literature review. Actas Urol Esp (Engl Ed) 46(8):481–486 36117081 10.1016/j.acuroe.2022.04.001

[23] Schneider M, Dinkelborg K, Xiao X, Chan-Smutko G, Hruska K, Huang D et al (2018) Early onset renal cell carcinoma in an adolescent girl with germline FLCN exon 5 deletion. Fam Cancer 17(1):135–139 28623476 10.1007/s10689-017-0008-8

